# Role of protein kinase CK2 in antitumor drug resistance

**DOI:** 10.1186/s13046-019-1292-y

**Published:** 2019-07-05

**Authors:** Christian Borgo, Maria Ruzzene

**Affiliations:** Department of Biomedical Sciences, University of Padova, Via U. Bassi 58b, 35131 Padova, Italy

**Keywords:** CK2, Drug resistance, Protein kinase inhibitors, Antitumor agents, DNA repair, Casein kinase 2, CKII

## Abstract

Drug resistance represents the major reason of pharmacological treatment failure. It is supported by a broad spectrum of mechanisms, whose molecular bases have been frequently correlated to aberrant protein phosphorylation. CK2 is a constitutively active protein kinase which phosphorylates hundreds of substrates; it is expressed in all cells, but its level is commonly found higher in cancer cells, where it plays anti-apoptotic, pro-migration and pro-proliferation functions. Several evidences support a role for CK2 in processes directly responsible of drug resistance, such as drug efflux and DNA repair; moreover, CK2 intervenes in signaling pathways which are crucial to evade drug response (as PI3K/AKT/PTEN, NF-κB, β-catenin, hedgehog signaling, p53), and controls the activity of chaperone machineries fundamental in resistant cells. Interestingly, a panel of specific and effective inhibitors of CK2 is available, and several examples are known of their efficacy in resistant cells, with synergistic effect when used in combination with conventional drugs, also in vivo. Here we analyze and discuss evidences supporting the hypothesis that CK2 targeting represents a valuable strategy to overcome drug resistance.

## Background

### CK2 structure, functions, and relevance to cancer biology

CK2 is a constitutively active acidophilic Ser/Thr protein kinase, usually present in cells in a tetrameric form, composed of two catalytic subunits (α or its isoform α’), and two regulatory subunits (β), with major functions in controlling substrate selectivity and enzyme stability [1]. It is expressed in all tissues of all eukaryotic organisms, and is essential for normal embryo development [2].

CK2 phosphorylates hundreds of substrates, involved in practically all cellular processes, but its main functions are related to cell growth, proliferation, and survival. Initial studies of down-regulation of CK2 expression in cells [3] or cell treatment with CK2 inhibitors [4] allowed to postulate the anti-apoptotic role of this kinase. Later, it was clear that the prevention of caspase action [5], but also the potentiation of different survival signaling and a multitude of other mechanisms, contribute to mediate a global anti-apoptotic function of CK2 [6, 7].

For a long time, CK2 has not been considered a convenient drug target, due to its ubiquity. However, suppression of apoptosis, and in general all CK2 functions, are especially important for cancer cells. CK2 was indeed defined as a key player in cancer biology [8] and proposed as a promising anticancer drug target [9]. Now, it is well accepted that cancer cells rely on CK2 activity more than healthy cells, in a sort of non-oncogene addiction [7]. Consistently, by the CRISPR/Cas9 technology, we could successfully produce non-tumor cells depleted of both CK2 catalytic isoforms [10], whereas no tumor cell completely devoid of CK2 activity was viable so far. Several in vitro studies with CK2 inhibitors have confirmed a higher sensitivity of tumor cells compared to normal counterparts, and animal treatments, as well as initial clinical trials in humans, are providing evidence of the feasibility of CK2 targeting for tumor therapy (see below, paragraph on CK2 inhibitors).

### General principles of tumor drug resistance

Chemotherapy is one of the major weapons against cancers, however, its therapeutic effectiveness is jeopardized by the intrinsic or acquired resistance to drugs, often displayed by cancer cells.

The mechanisms underlining drug resistance are multiple and only partially known. Several reviews deal with them, e.g. [11–13], to whom the readers are referred to. Here we just mention that the major ones are the reduction of intracellular drug concentration (due to drug efflux or drug metabolism), the mutation or altered expression of the drug target, and DNA damage repair mechanisms. In addition to these events, which are specifically responsible of resistant phenotypes, other processes are related to reduced responses to antitumor agents, such as downstream mechanisms of survival (reduced apoptosis, autophagy, necroptosis), and adaptive changes, due to redundant pathways, epithelial-mesenchymal transition (EMT), and to the protective effect of microenvironment. Among the signaling pathways relevant in this adaptive rewiring, special interested is given to the PI3K/AKT/mTOR signaling, the activity of the HSP90 machinery, and the hypoxia. In most of these listed crucial events for the onset of drug resistance, functions for CK2 have been reported, as it will be described in each specific paragraph.

## Main text

### CK2 and drug resistance in cancer cells

CK2 is an anti-apoptotic kinase, which sustains cell survival by several mechanisms (see above). In addition to its function in protecting cells from the cytotoxic effect of antitumor drugs, its role has been also specifically described in the background of drug resistance, either in processes directly responsible for resistance, such as drug efflux and DNA repair, or in signaling pathways which are crucial to evade drug response and are fundamental in resistant cells. Consistently, in a proteomics study aimed at comparing the phosphorylation stoichiometry in drug-sensitive and resistant lung cancer cells, a huge number of differently phosphorylated putative CK2 substrates was found [14]. The paragraphs below describe each different level of the CK2/drug resistance connection. A list of proteins implicated in drug resistance whose phosphorylation and/or expression level is controlled by CK2 is reported in Table 1.

**Table 1:** Major drug resistance-related proteins which have been reported as regulated by CK2 (via direct phosphorylation and/or control of the protein amount)

Protein	Function	Phospho-site	Phosphoryl. by CK2	Protein level controlled by CK2	REF
ABCG2	MDR efflux pump			Yes	[15]
AKT (PKB)	Ser/Thr kinase	Ser129	Yes		[16–18]
ARC	Apoptosis repressor	Thr149	Yes		[19]
BRD4	Epigenetic regulator and transcription cofactor		Yes		[20]
CDC37	Co-chaperone protein	Ser13	Yes	Yes	[21]
EGFR	Receptor tyrosine kinase			Yes	[22]
FLIP	Inhibitor of apoptosis			Yes	[23]
GLI2	Transcription factor			Yes	[24]
HMGA1	DNA replication, transcription, heterochromatin organization		Yes		[25]
HSP27	Chaperone protein			Yes	[26, 27]
HSP70	Chaperone protein			Yes	[26]
HSP90	Chaperone protein	Thr22 Ser225 Ser254	Yes		[28, 29]
IKBα	NF-κB inhibitor	Ser32 Ser36	Yes		[30]
MDC1	DNA repair (DSB)		Yes		[31]
MRE11	DNA repair (DSB)		Yes	Yes	[32]
MRP1	MDR efflux pump	Thr249	Yes		[33]
NF-κB p65(RelA)	Transcription factor	Ser259	Yes		[22, 30, 34–36]
p53	Tumor suppressor	Ser392	Yes	Yes	[37–39]
P-gp	MDR efflux pump	Ser665 Ser669 Ser681	Yes	Yes	[28, 40]
PTEN	Lipid phosphatase	Ser370, Ser380, Thr382, Thr383, Ser385 (*)	Yes	Yes	[41–43]
STAT3	Transcription factor	Ser727	Yes		[36]
Survivin	Inhibitor of apoptosis			Yes	[44]
TAp73	Tumor suppressor	Thr27	Yes	Yes	[45]
Topoisomerase I	DNA structure and function regulation	Ser506	Yes		[46–48]
Topoisomerase II	DNA structure and function regulation	Ser1106	Yes		[49–52]
XRCC1	DNA repair (SSB)	S485/T488 and S518/T519/T523	Yes		[53–55]
XRCC4	DNA repair (DSB, NHEJ)	Thr233	Yes		[56]

The quotations refer to the publications reporting a CK2-dependent regulation of these proteins in a drug resistance context. (*) Sites are reported in ref. [57]

#### CK2 and drug efflux

An increased drug efflux is a common event in the multidrug resistance (MDR), a phenotype displayed by cells which become insensitive to a broad range of cytotoxic agents. In these cases, the (over) expression of a pump mediating extrusion of chemotherapeutic drugs from the cell is observed. These pumps are proteins belonging to the ABC family. In humans, three of them are known to mediate MDR: the P-glycoprotein (P-gp, also known as MDR1/ABCB1), the MDR-associated protein (MRP1, ABCC1), and the breast cancer resistance protein (BCRP, ABCG2) [12].

Several evidences support a role of CK2 in the regulation of MDR pumps activity. In 2007, we demonstrated that inhibition of CK2 allowed an increased accumulation of doxorubicin in P-gp expressing cells [58]. Although a direct evidence of a cause/effect relationship of phosphorylation on P-gp activity is still lacking, P-gp is indeed known as a substrate of CK2 [40]; since our results were obtained in cell treated with CK2 inhibitors for a very short time (30 min), they strongly suggest a direct regulation due to phosphorylation. Additionally, more recently CK2 was found to increase drug-induced P-gp amount, through a mechanism involving the phosphorylation of heat shock protein 90β (HSP90β) and subsequent stabilization of Pregnane X receptor (PXR), which dissociates and translocates into the nucleus to interact with RXR (retinoid X receptor) and induce the transcription of *ABCB1* gene [28].

A regulatory role of CK2 was later found also on MRP1: a model was proposed in which CK2 potentiates MRP1 function through direct phosphorylation of Thr249; in fact, CK2α knock-down, or MRP1 Thr249Ala mutation, decreased the efflux of doxorubicin and increased doxorubicin cytotoxicity in MRP1-expressing cells [33]. The expression level of *ABCG2* was found dramatically decreased in CK2α-silenced lung cancer cells, due to down-regulation of the hedgehog signaling (see below for the CK2 impact on this signaling) [15].

**Fig. 1:**
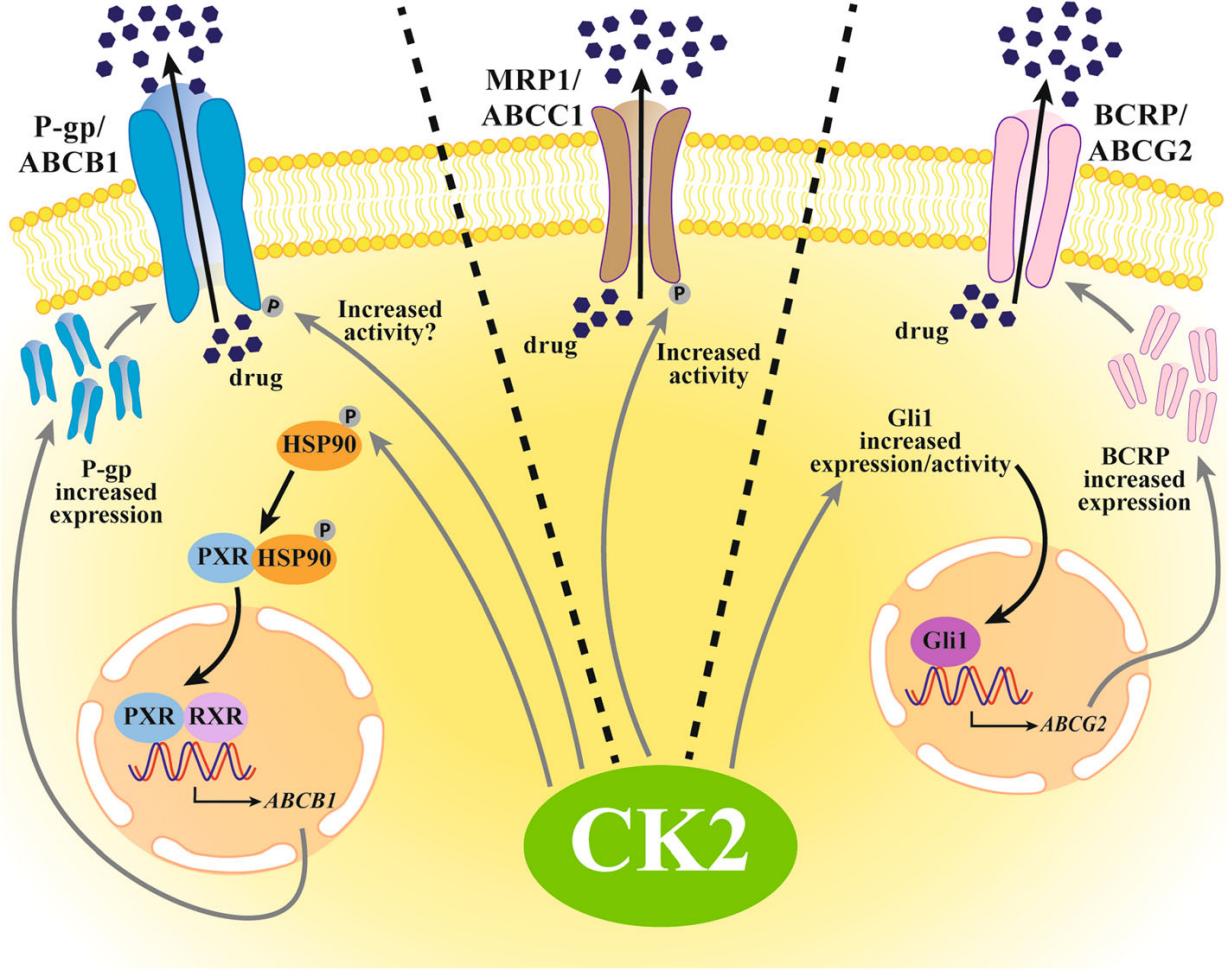
CK2-dependent control of drug efflux through actions on the MDR pumps

Summarizing, CK2 not only phosphorylates P-gp, with possible consequences on its extrusion activity, but also induces its overexpression, thus amplifying the MDR phenotype; it phosphorylates and activates MRP1, the other major extruding pump mediating MDR, and controls the expression of the third ABC family extrusion pump (BCRP) (Fig. 1).

Our initial studies on the CK2 roles in MDR cells disclosed a possible specific function of the monomeric CK2 α isoform, since the MDR cells analyzed (CEM-R) expressed a higher level of CK2 α, but not CK2 β, compared to their parental line [58]. However, this does not seem to be a general feature of MDR cells, since a subsequent analysis revealed a variable scenery of CK2 expression in resistant compared to sensitive cells [59]. Regardless of the overexpression of CK2 (or of a specific CK2 isoform), the general observation is that MDR cells are sensitive to different kind of CK2 inhibitors, as demonstrated by our and other groups [22, 59, 60] (see also the paragraph on CK2 inhibitors), suggesting that MDR pumps are not active towards chemical compounds targeting CK2.

On this regard, it is worth to mention that CK2 inhibitors have been derivatized for targeting both CK2 and the breast cancer resistance protein ABCG2 [61]. The same group found that the structure-activity relationships for CK2 and ABCG2 are totally different, and they developed compounds blocking the extrusion pump without significantly inhibiting CK2 [62]; however, a dual CK2/ABCG2 inhibitor is particularly interesting, considering that co-administration of pump-inhibitors and cytotoxic agents is one of the strategies proposed to fight MDR [13].

#### Drug resistance and CK2-mediated DNA repair

**Fig. 2:**
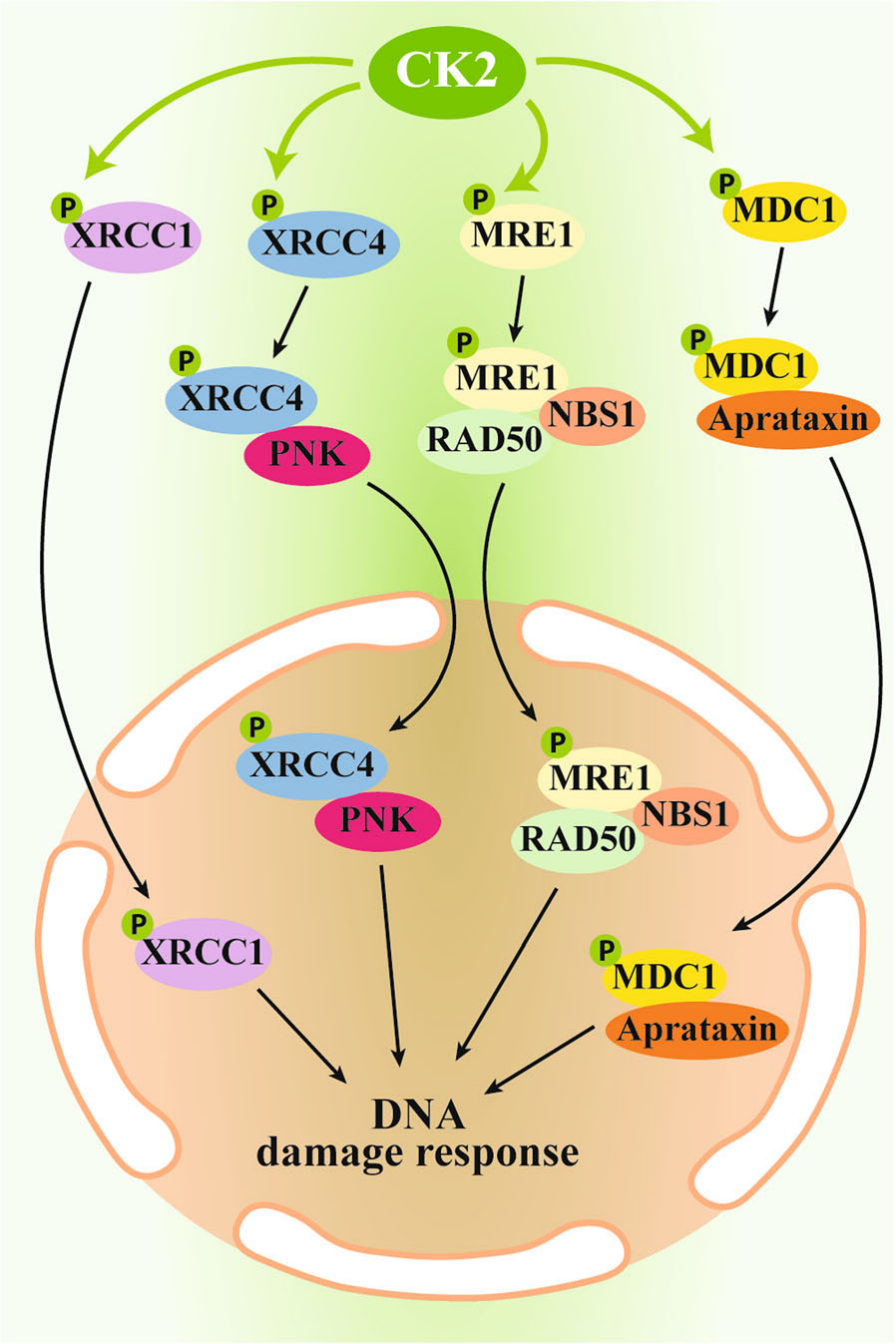
Mechanisms of CK2 control on cellular responses to DNA damage in chemo-resistant cells

DNA damage is a direct or indirect event in response to many antitumor agents, and a major mechanism that cells develop to evade their effects is an increased DNA repair activity. CK2 is a key player in the cellular response to DNA damage. Its role in phosphorylating the scaffold protein XRCC1, thus promoting DNA single-strand break repair, is known for several years [63]. CK2 phosphorylates also XRCC4 [56], a crucial protein for the nonhomologous end-joining (NHEJ), the major DNA double-strand break (DSB) repair pathway. The phosphorylation of XRCC4 at Thr233 by CK2 is required for its association to PNK, which is essential for optimal DSB repair (and indeed a not-phosphorylatable XRCC4 mutant displayed increased sensitivity to radiation-induced DNA-damage). Many other proteins implicated in DNA repair were later found to be regulated by CK2, such as the heterochromatin protein 1 (HP1) β [64], the DNA damage mediator protein MDC1 [65], the DNA-dependent protein kinase [66], the recombinase Rad51 [67], the deubiquitylase OTUB1 [68], the adaptor protein 53BP1 [69], the MLH1 component of the DNA mismatch repair complex MutLα [70], and the yeast proteins Lif1 (regulatory subunit of the NHEJ-specific DNA ligase IV) [71] and Ctp1 (DNA end-processing factor) [72]. Consistently, the specific CK2 inhibitor CX-4945 (also known as silmitasertib) has been shown to suppress the DNA repair response to anticancer drugs [73]. The global role of CK2 in DNA damage response and repair pathways has been recently reviewed [74]. To the purpose of this review, we focus on those publications where the CK2 action on DNA repair has been found specifically responsible of cancer drug resistance (Fig. 2**)**. In particular, a body of evidence indicates a role of XRCC1-CK2 axis in the resistance to Cisplatin and derivatives. Pt-based compounds are DNA-damaging agents; they are not substrate of P-gp, MRP1 and ABCG2 (they can be exported by other transporter such as the copper efflux transporter, ATP7A and ATP7B [12]), and a main mechanism of resistance to these drugs is an increased DNA repair. Consistently, a protective role of CK2 has been frequently reported. For example, it has been shown that lung fibroblasts from idiopathic pulmonary fibrosis (IPF) patients is due to CK2 hyperactivation, which in turn promotes an abnormally high XRCC1 activity. In fact, CK2 blockade sensitizes IPF fibroblasts to Cisplatin [53]. Moreover, the phosphorylation of XRCC1 by CK2 is required for the action of its regulator JWA in gastric cancer cells resistant to DNA repair, following Cisplatin-induced DSBs [54]. Interestingly, conjugated compounds, with a CK2-inhibiting moiety linked to platin-derived drugs, reverse Cisplatin resistance in cancer cells by suppressing DSB repair by CK2 [30, 32]. One of the conjugated compound has been found effective in reversing drug resistance also in vivo, with a molecular mechanism involving the DSB repair MRE11-RAD50-NBS1(MRN) complex [32]. A similar approach of chimeric agent has been exploited to produce a Cx-platin drug, a CK2-targeting Pt (IV) prodrug, containing the CK2 inhibitor CX-4945 [31]. It is effective in suppressing CK2-mediated DNA damage repair and reversing Cisplatin resistance. The identified mechanism was the prevention of MDC1 phosphorylation by CK2 and its association to the FHA domain of aprataxin at the DSBs. In vivo studies showed a higher antitumor efficacy of Cx-platin compared to Cisplatin [31].

Another in vivo study showed a synergistic effect of Cisplatin used in combination with the CK2-targeting compound CIGB-300, in nude mice xenografted with human cervical tumor cells, and an increased mice survival compared to single-agent treatment was observed [75].

The rationale of targeting CK2 in combination with Cisplatin-based compounds is also supported by the observation of an increased CK2 expression in response to these drugs: this was reported by Yang et al. [76], who observed down-regulation of the tumor suppressor PML in cisplatin-treated lung cancer cells, as a consequence of CK2α overexpression.

The multitude of CK2 targets implies a network where the kinase simultaneously intervenes at different levels in mediating the response to DNA damaging agents. An example is provided by Kang and coworkers showed that XRCC4, PTEN and p53 (that are all CK2 substrates) merge on a concerted signal, which produce resistance to the DNA damage-inducing drug doxorubicin in glioblastoma cells. Sensitivity can be restored by restraining CK2, and the authors suggest that combinatorial therapies based on CK2 targeting might potentially counteract therapeutic resistance in glioblastoma patients with aberrancies in p53, PTEN and CK2 [41].

Topoisomerase inhibitors are a class of antitumor agents with final effects on DNA integrity and functions. CK2 is deeply implicated in the functionality of both topoisomerase I and II; however, since this is not a pure matter of CK2 control on DNA repair, and given the complexity of the issue, it will be dealt below, in a specific paragraph.

#### CK2 control on chaperone machinery in drug resistance

Chaperone proteins are often expressed at high levels in tumors and closely associated with a poor prognosis and resistance to therapy [77]. CK2 is considered a master regulator of chaperones [27, 78, 79], by which it exerts its protective function on onco-kinases and other survival proteins. In this sense, the CK2-dependent protection from apoptosis mediated by the chaperone machinery is obvious. More specifically focusing on drug resistance, it has been found that acquired MDR in response to rifampin treatment is correlated to the phosphorylation of HSP90β at Ser225 and Ser254 by CK2: phospho-HSP90β forms a more stable complex with the Pregnane X receptor (PXR), the transcription factor of the P-gp gene (MDR1), and this explains the final induction of *ABCB1* expression due to CK2 [28].

Moreover, it has been observed that CK2 inhibition reduces the association between HSP90 and the co-chaperone CDC37 in cancer cells resistant to conventional therapies, with a consequent down-regulation of HSP90-client proteins (EGFR, PTEN, mTOR, Raptor and Tuberin/TSC2) [22].

Targeting chaperones belonging to the family of heat shock proteins (HSP) is a promising antitumor strategy, since many of their clients are involved in tumor development and progression [77]. In particular, several HSP90 inhibitors are in clinical trials [80], but different factors may influence cellular susceptibility to them, and resistance can occur due to redundant pathways or increased levels of other pro-survival chaperones in response to prolonged treatment. Therefore, combination therapies are considered the more promising approach to prevent compensatory mechanisms [80]. Consistently, the co-treatment of glioblastoma cells with a CK2 inhibitor (D11) was found effective in preventing the increase in HSP70 amount in response to the HSP90 inhibitor 17-AAG, and concomitantly also a reduction of the co-chaperone HSP27 was observed [26].

A different story has been found by a yeast based assay: it has been shown that the phosphorylation of Thr22 in yeast HSP90 by CK2 regulates the chaperone function, but increases the sensitivity to HSP90 inhibitors in vivo [29]. Results have been produced in a yeast strain devoid of the drug resistance pump PDR5, the major mediator of HSP90 inhibitor efflux. Although they need to be confirmed in mammals, they suggest caution against too cursory generalization of CK2 targeting as a sensitization strategy towards HSP90 inhibitors.

Also HSP27 inhibitors are considered valuable tools against drug resistance [81], and we have recently found that CK2 strictly controls the turnover of this chaperone in cervical and liver cancer cells [27].

The co-chaperone CDC37, which has a prominent role on the onco-kinome, is also controlled by CK2 [79]. The suppression of CDC37 phosphorylation and stabilization by CK2 was recently identified in the mechanism exploited by the microtubule-targeting pyrrolo-1,5-benzoxazepine compound for overcoming resistance to imatinib of gastrointestinal stromal tumor cells [21].

**Fig. 3:**
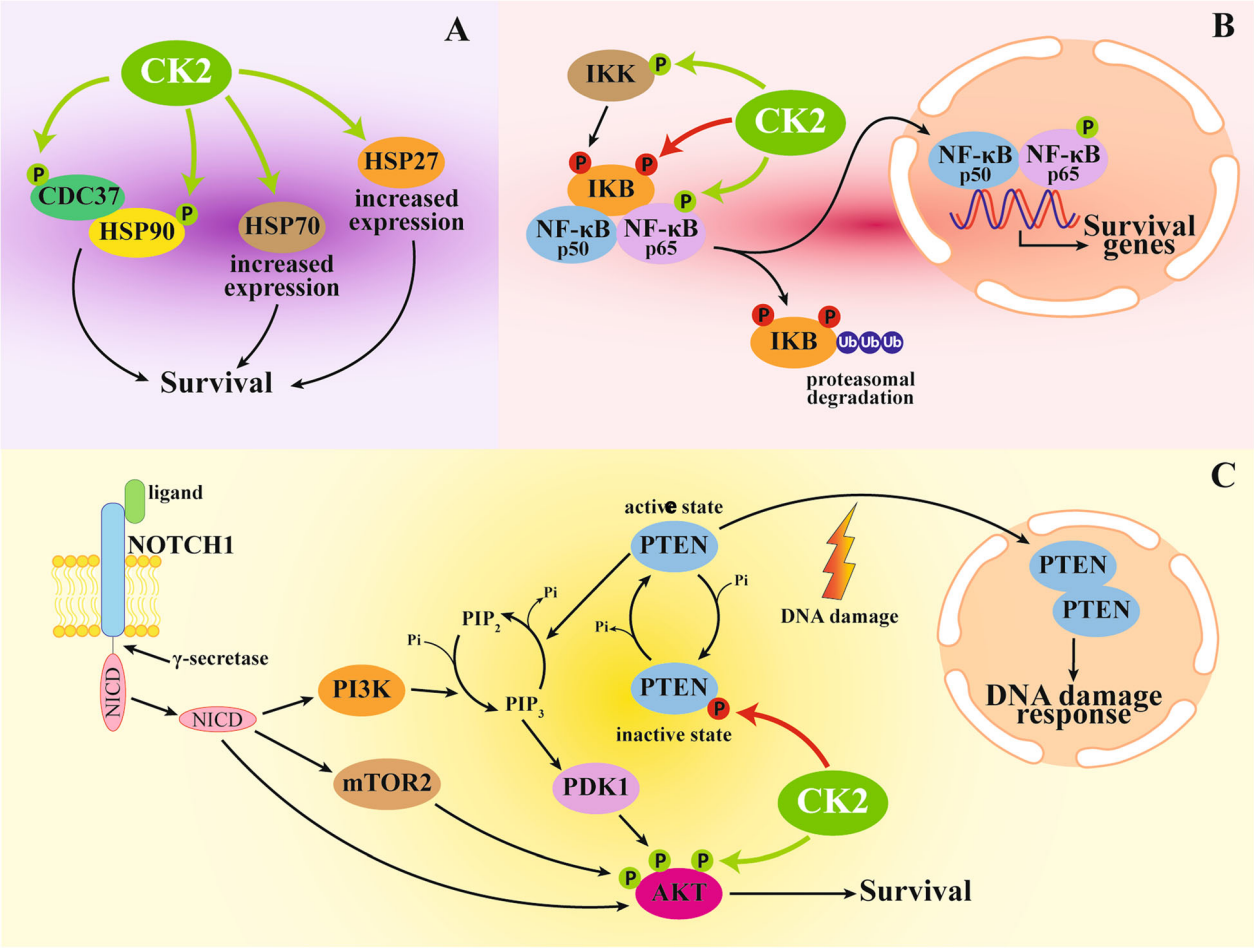
CK2 actions on chaperone machinery (**a**), NF-κB signaling (**b**), and PI3K/AKT/PTEN pathway (**c**). Phosphorylation causes substrate inhibition if indicate in red, activation if indicated in green

Figure 3a summarizes the major chaperone proteins controlled by CK2.

#### CK2 control on signaling pathways mediating escape to drug response

CK2 has been widely implicated in survival signaling (as reviewed in [7]), and it represents a target of general validity to downregulate different hyperactive pathways that can cause abnormal survival of cancer cells. Here below, we separately describe the pathways where the intervention of CK2 has been found crucial for apoptosis resistance.

##### NF-κB signaling

This transcription factor positively modulates the expression of several survival genes; its regulation by CK2 is known for many years (as reviewed in [7]), due either to the prevention of IKB mediated inhibition, or to the direct phosphorylation of p65 subunit (Fig. 3b). On this latter, the major CK2-site is Ser529 (Wang et al., 2000), whose diminished phosphorylation in response to CX-4945 (with consequent attenuation of NF-κB activity) was described as instrumental for restoring sensitivity to anti-androgens of castration-resistant prostate cancer cells [34]. Another study showed that the potentiation of NF-κB signaling by CK2, mediated by p65 phosphorylation at Ser529, is the crucial event to increase resistance to the proteasome inhibitor bortezomib [36], also providing evidence for the importance of Ser727 STAT3 phosphorylation by CK2.

The suppression of NF-κB activity was also reported as major mechanism by which down-regulation of CK2 sensitizes prostate cancer cells to the antitumor effect of TRAIL; however, in this case the crucial p65 phosphorylation site was Ser536 [35]. This is a site of alternative NF-κB activation [82], not directly targeted by CK2, which however might represent an integrator for multiple signaling pathways.

TNFα is a pro-apoptotic agent, but several tumors are resistant to its effect, and this is attributed to the activation of NF-κB signaling. In glioblastoma cells resistant to TNFα, CK2 inhibitors have been found to restore sensitivity by abrogating NF-κB activation [39].

Other studies on CK2 in cells resistant to apoptosis support the implication of the NF-κB pathways: its attenuation has been considered a major mechanism for the reversal of Cisplatin resistance induced by CK2-blockade [30], and it has been demonstrated to be reduced, together with EGFR expression, in CK2-inhibitor treated glioblastoma and pancreatic adenocarcinoma cells resistant to conventional chemotherapy [22].

However, it is worth to mention that a variability of the NF-κB response to CK2 inhibitors was noticed [83], and a delayed activation of NF-κB has been suggested as correlated with resistance to CK2 inhibitors in malignant gliomas [83]. This observation highlights the complexity of the problem, where NF-κB covers a double function of allowing response and inducing resistance to CK2 inhibitors. Further studies will be necessary to confirm whether NF-κB activation may elicit resistance to CK2 inhibitors, in which case the feasibility of combined CK2/NF-κB targeting should be evaluated.

##### PI3K/AKT/PTEN signaling

CK2 is deeply interconnected with this survival pathways, that it boots with interventions at several levels (as reviewed in [84]). PTEN is a major site of action for CK2 in this signaling axis: it is regulated by a counterintuitive mechanism in which phosphorylation by CK2 increases its protein amount but decreases its lipid phosphatase activity. In p53-deficient glioblastoma tumors resistant to DNA-damaging agents, a crucial role has been found for CK2 also in PTEN localization: upon DNA damage, PTEN fails to accumulate in the nucleus, and is retained in the cytoplasm in its monomeric inactive state, due to its phosphorylation by CK2. CK2 inhibition restores PTEN nuclear distribution, and the consequent DNA damage signaling cascade required for the response to drugs [41]. The reactivation of PTEN in response to CK2 blockage was the identified mechanism by which the inhibitor TBB (4,5,6,7-tetrabromobenzotriazole) promotes apoptosis in CML (chronic myeloid leukemia) cells from imatinib resistant patients [85].

In a system biology study aimed at identifying the signaling network underlining the sensitivity-to-resistance transition in response to HER2 inhibition, CK2 has been identified, in virtue of its regulation of PTEN, as responsible of a compensatory mechanism in case of vulnerable mutations [42].

Pharmacological inhibition of NOTCH1 with γ-secretase inhibitors (GSIs) is a promising therapeutic strategy against several tumors, especially T-ALLs, which present NOTCH1 activating mutations in over 50% of cases [86]. However, GSI-resistance often occurs for several reasons; among them, PTEN loss is a major one. Since in a substantial fraction of T-ALLs PTEN is expressed, but is inactive due to CK2-mediated phosphorylation, it is suggested that GSI clinical efficacy can be improved by inhibiting CK2 [87]. In fact, CK2 inhibitors have already been reported to synergize with GSIs [88].

The CK2/PTEN axis is also implicated in the response to BRAF inhibitors. These compounds are therapeutic tools for tumors harboring *BRAF* mutations producing a constitutive active kinase; however, as with many other targeted therapies, acquired resistance frequently occurs in response to treatment. Recently, it has been shown that, in melanoma cells, chodroitin-4-sulfate confers resistance to BRAF inhibitors by a mechanism involving an increased CK2/PTEN binding, with consequent PTEN inhibition [43]. In another study, the reduction of AKT signaling has been claimed to explain the lethal synergism of melanoma and thyroid carcinoma cotreatment with BRAF and CK2 inhibitors [89], but, in this case, PTEN was unchanged in cells treated with the CK2 inhibitor, suggesting a different level for the action of CK2 on this pathways. Interestingly, the authors clearly showed that the *BRAF* lesion was required for CK2 synergism to be effective: in cells expressing wild type (wt) BRAF, minor or even antagonistic effects were observed. It is worth to notice that, regarding the CK2/BRAF nexus, a study demonstrated that the knock-down of CK2 in BRAF mutant melanoma cells was indeed accompanied by increased sensitivity to RAF-MEK inhibitors (with downstream effect on ERK phosphorylation); however, the authors proposed a kinase-independent scaffolding function of CK2, since the resistance to RAF-MEK inhibitors was promoted by overexpression of a CK2 kinase-inactive mutant [90].

Very recently, an in vitro and in vivo study showed CK2-dependent regulation of PI3K/AKT pathway in gastric cancer cells resistant to paclitaxel [18].

Several other studies have shown a reduced PI3K/AKT signaling in a drug resistance background (see e.g. [16, 17]), where AKT phosphorylation at Ser129 was used as a reporter of CK2 activity; although in these cases a direct correlation between the CK2 action of this pathway and the occurrence of resistance was not established, it is very likely that it significantly contributed to cell survival to treatments.

Figure 3c summarizes the multiple levels of CK2 intervention on PI3K/AKT/PTEN pathways which produces drug resistance.

##### p53

The p53 tumor suppressor is a debated CK2 substrate [37], and, in particular, its mediation of CK2 functions on drug resistance is quite contradictory. p53 was found crucial for the increase of daunorubicin effect induced by CK2 inhibition in acute myeloid leukemia cells [38]; similarly, the sensitization to TNFα induced by CK2 blockade in glioblastoma cells is mediated by p53 function activation [39]. Kang and coworkers [41] demonstrated that, in DNA damage-resistant glioblastoma cells, the cytoplasmic PTEN retention provoked by CK2 (see above) may be enforced by p53 deficiency, and suggested that p53 inactivation is a prerequisite to CK2 effects on PTEN. However, CK2 inhibition was found effective in malignant glial tumors, without any restriction to the p53 status [83]; this was in agreement with a study that exploited isogenic colon adenocarcinoma cell lines differing in the presence or absence of p53 to demonstrate that the enhancement of Apo2L/TRAIL-induced apoptosis by CK2 inhibitors is independent of p53 [91].

These contradictory results highlight the complexity of this issue. It is conceivable that, when several factors contribute to a global effect, it is hard to unequivocally distinguish the instrumental events from the collateral ones; it also possible that important differences occur depending on the cellular model considered, and clarification is needed in this regard. In any case, these findings suggest that *TP53* mutations/deletions should not be neglected in predicting responsiveness to CK2 inhibitors.

##### β-Catenin/survivin

Survivin (also called BIRC5) is a small protein belonging to the inhibitor of apoptosis protein family, and its aberrant expression in tumors confers resistance to drug-induced apoptosis [92]. CK2 is known to increase *BIRC5* expression via β-catenin-TCF/LEF-mediated transcription [93]. Therefore, CK2 targeting would prevent also this commonly observed survival signal.

##### Sonic hedgehog (SHH) signaling

Tumor driven by SHH are particularly aggressive and frequently resistant to SHH inhibitors. In a study [24] aimed at identifying novel putative therapeutic targets for SHH-dependent medulloblastomas, CK2 was found as a SHH signaling driver, and its inhibition was shown to decrease the viability of patient-derived cells resistant to SHH inhibitors, in vitro and in murine models. The crucial protein identified was GLI2, a late downstream effector in SHH signaling, which is stabilized and activated by CK2.

#### CK2 and the resistance to tyrosine kinase inhibitors (TKIs)

TKIs have been widely employed as antitumor drugs, since the deregulated activity of tyrosine kinases is among the most frequent causes of tumors [94]. However, the occurrence of resistance is often observed, due to different mechanisms, frequently represented by gene mutations or amplifications, but also by compensatory signaling [95]. Among the pathways which contribute to TKI failure, CK2 has been reported. We have found that, in imatinib-resistant CML cell lines, without *BCR-ABL1* mutations, CK2 differently potentiates the resistant phenotype, either by overexpressing its protein-level [17] or by a BCR-ABL-independent activation of rpS6 [16]. A proteomic study in non-small-cell lung cancer cells identified CK2 as a central element to mediate TKI resistance, and the phosphorylation of its substrate HMGA1 as a critical node to promote resistance to the EGFR inhibitor gefitinib [25]. CDC37 is another CK2 substrate critically important in TKI resistance: in imatinib-resistant gastrointestinal stromal tumors (GIST) cells with constitutively active c-KIT receptor tyrosine kinase, a mechanism has been described where CK2 has a role through the phosphorylation of CDC37, which in turn, in a positive loop, contributes in maintaining high levels of CK2 itself. In fact, treatments with PBOX-15 (pyrrolo-1,5-benzoxazepine, a microtubule-targeting compound), which reduce both CDC37 and CK2 levels, increase sensitivity to imatinib [21]. In CML bone marrow samples derived from patients resistant to imatinib, due to BCR-ABL T315I point mutation, a pro-apoptotic effect of CK2 inhibition was reported, mediated by PTEN reactivation [85].

In EGFR mutant lung cancer cells resistant to TKIs, CX-4945 was reported to sensitize cells and induce autophagy; however, cell recovery was observed upon CX-4945 withdrawal [96]. This is in accordance with our previous finding on CX-4945 short persistence [97], and suggests that this feature should be considered in planning protocols for in vivo treatments with CX-4945. A further note of caution comes from a study where a different CK2 inhibitor, Quinalizarin, was exploited to reduce viability, proliferation and migration of lung adenocarcinoma cells with different EGFR genotypes [98]. The authors found variability in the responses, according to the mutation harbored, and concluded that the effects were especially evident in cells harboring TKI sensitive EGFR mutations; actually, one of the TKI-resistant mutant cell lines was almost insensitive to Quinalizarin in an MTT viability assay. The reasons of this different sensitivity to Quinalizarin were not analyzed, neither other CK2 inhibitors were tested; however, this study suggests speculations on compensative mechanisms possibly occurring, in a background of TKI resistance, which imply resistance also to CK2 inhibition, and will deserve future investigation. Similarly, an only modest effect of CK2 inhibition was recently reported in KRAS-active non–small cell lung cancer cells resistant to EGFR inhibitors [99].

Interestingly, CK2 can regulate also the expression of EGFR itself, as shown by its down-regulation in response to CK2 inhibition [22].

Regardless of sensitive or resistant phenotype, several studies observed synergistic effects of CK2 inhibitors and TKI in tumor cells. Among them, Bliesath and colleagues, by combining the CK2 inhibitor CX-4945, and the EGFR tyrosine kinase inhibitor erlotinib, observed a synergistic antitumor effect in non-small cell lung carcinoma and squamous cell carcinoma, in vitro and in vivo, and demonstrated that it was mediated by an enhanced attenuation of the PI3K/AKT/mTOR pathway [100]. Similarly, an in silico study suggested that the PI3K/PTEN/AKT pathway could be synergistically reduced by simultaneous targeting of the receptor tyrosine kinase HER2 and CK2 [42]. Very recently, a study in KRAS-active non–small cell lung cancer cells resistant to EGFR inhibitors reported that single treatment with a CK2 inhibitor was not sufficient to completely impair cell viability, and an informatic analysis revealed MEK as a possible second target to overcome resistance; indeed, CX-4945 in combination with the MEK inhibitor AZD6244 displayed synergistic effects [99].

In summary, CK2 targeting is widely suggested as a potential therapeutic strategy for improving the response to TKI, although the issue might deserve a deeper investigation.

### The topoisomerase I and II issue

Topoisomerases (topo) are crucial enzymes for the maintenance of genomic integrity. Consequently, topo I and II are targets for widely used antitumor drugs; however, resistance frequently occurs, by mechanism still incompletely known, not simply ascribable to reduced drug accumulation [101]. Both topo I and II are known substrates of CK2 (see Table 1), and indeed their CK2-dependent phosphorylation has been considered as related to resistance to topo-targeting drugs. The topic deserves special attention, since reported results are somehow counterintuitive.

As far as topo II is concerned, its phosphorylation by CK2 and other kinases is known for many years, and it has been reported as crucial to alters enzyme activity and sensitivity to drugs, such as etoposide (VP-16) [50]. Conversely, the importance of the distinct phosphorylation sites is less clear [101]. Topo II Ser-1106 phosphorylation, initially hypothesized as due to CK2, was found to positively modulate etoposide sensitivity [49]; later, the same group demonstrated that CK1 delta/epsilon, and not CK2, is responsible of this phosphorylation [102]. Consistently, no overexpression of CK2 was found in cells displaying hyperphosphorylated topo II [52]. A study of ectopic expression of yeast topo II mutated at different CK2 phospho-acceptor sites reported of not altered sensitivity to etoposide [51]. Summarizing, we can conclude that, despite the strong evidence of CK2-dependent phosphorylation of topo II, no clear-cut effect on drug sensitivity has been assigned to CK2, so far.

The CK2/topo I connection seems to be a quite different story. Topo I is targeted by camptothecin and derivatives (as Irinotecan and Topotecan). CK2 phosphorylates topo I at Ser506, and this enhances topo I-DNA binding and cellular sensitivity to camptothecin [47]. Indeed, a correlation was found between low levels of CK2 and resistance to topo I inhibitors, and CK2 has been suggested among possible biomarkers of therapy-responsive tumors [48]. In agreement, in a study on camptothecin sensitive and resistant subpopulations of colorectal cancer cells Caco2, it was proposed that CK2 can convert topo I from a resistant to a sensitive form [46]. Altogether, these findings suggest an unusual and counter-trend role of CK2 in the resistance to camptothecin and derivatives, where this pro-survival and antiapoptotic kinase promotes drug sensitivity instead of resistance.

### CK2 in cancer stem cells: relevance to drug resistance

CK2 has emerged as a possible regulator of cancer stem cell (CSC) genes [103, 104]. Down-regulation of CK2β in epithelial cells induces the acquisition of stem cell-like properties [105], and CK2 inhibition significantly affects the neural stem cell niche [106]. A major role of CK2 has been found in the functions of glioblastoma brain tumor initiating cells (BTICs) [107]. Furthermore, CK2 is positively involved in hedgehog signaling, which is important in stem cell maintenance, and inhibition of CK2 has been proposed to reduce stem-like side population in human lung cancer [15].

A crucial target for the CK2 function in promoting the expression of CSC genes has been identified in the TAp73 tumor suppressor, which is phosphorylated and inhibited by CK2 [45]. Recent findings highlight an altered CK2 amount in a quantitative proteomic analysis of CD34^+^ cells from CML patients treated with a pro-apoptotic inhibitor [108]. In acute myeloid leukemia stem cells, CK2 targeting was found to induce accumulation in the late S-G2-M phases, trigger apoptosis, and increase sensitivity to doxorubicin [109]. The study suggests CK2 as a therapy target to minimize the persistence of residual leukemia cells.

CSCs are strongly related to therapeutic resistance, since they inherit the ability to inactivate cytotoxic drugs by a number of different mechanisms [110]; therefore, the finding that CK2 inhibitors are effective also against this cell niche has great relevance from a therapeutic point of view.

### CK2 inhibitors, alone and in combined treatments, as a strategy to overcome drug resistance

A huge number of ATP-competitive CK2 inhibitors have been developed so far, many of them displaying significant selectivity due to the peculiar features of the CK2 ATP pocket [111, 112]. The most promising compound is CX-4945 [113], presently in clinical trial for different cancers (https://clinicaltrials.gov/ct2/results?cond=&term=cx-4945&cntry=&state=&city=&dist=).

In 2007, we published that a number of CK2 inhibitors displayed similar effectiveness in inducing apoptosis in drug sensitive and resistant leukemia cells [58]. Later, we demonstrated that CX-4945 and its analog CX-5011 are effective in inducing apoptosis in several types of drug resistant cells [60]. Since then, several other reports have confirmed the possibility to exploit CK2 inhibitors against drug resistance. CX-4945 could restore the sensitivity of castration resistant prostate cancer cells (CRPC) to bicalutamide [34]; DMAT (2-dimethylamino-4,5,6,7-tetrabromobenzimidazole) and TBBz (4,5,6,7-tetrabromo-1H-benzimidazole) produce an increased doxorubicin accumulation in MRP-1 expressing cancer cells [33]. DMAT was also used against human breast cancer cells with acquired resistance to antiestrogens (while it fails to kill parental cells, due to their higher level of Bcl-2) [114], and D11 (1,3-Dichloro-6-[(E)-((4-methoxyphenyl)imino)methyl] diben-zo(b,d) furan-2,7-diol) induced apoptosis and impaired cell migration in glioblastoma and pancreatic cancer cell lines resistant to conventional chemotherapeutic agents [22]. Martins and coworkers [115] reported on the efficacy of CX-4945 in chronic lymphocytic leukemia (CLL) lines and primary cells from patients resistant to the purine analog fludarabine; the more sensitive samples were those with a shorter lymphocyte doubling time, therefore the authors hypothesize that patients with advanced-stage disease may especially benefit from CX-4945 treatment. Moreover, because stromal support may contribute to leukemia drug resistance, they assessed the pro-apoptotic effect of CX-4945 in CCL co-cultures, and found that it was not significantly reversed by stromal cells. Very recently, the inhibition of CK2 was demonstrated to overcome the resistance to paclitaxel in gastric cancer [18]: CX-4945 exhibited synergistic effect, in combination with paclitaxel, in reducing tumor growth in a xenograft murine model.

Interestingly, TBB and CX-4945 were successfully used against medulloblastoma cells derived from patients resistant to vismodegib (a hedgehog signaling inhibitor); responsiveness was observed both in vitro and in xenograft mouse models, with a significative extension of the survival of treated tumor-bearing mice [24].

Derivatives of pyridocarbazole and benzopyridoindole are other ATP-competitive CK2 inhibitors displaying in vitro and in vivo antitumoral activity in p53 mutant glioblastoma cells particularly resistant to drug-induced apoptosis [116]. In the same cell line, allosteric inhibitors of CK2 have been also proven to be effective [117]. Another non-ATP competitive compound, CGIB-300, was found to modulate the expression level of proteins implicated in the chemotherapy resistance in non-small cell lung cancer cells [118]. The dual inhibitor TDB, hitting the pro-survival kinase Pim1 in addition to CK2, was also exploited to kill MDR cells [119]. This compound was even more efficient than CX-4945, consistently with the concept of the “non-selective selectivity”, by which the controlled inhibition of a small panel of enzymes might be convenient, in order to prevent possible compensation events.

Similarly, the combination of a kinase inhibitor with a conventional drug is presently considered a successful strategy to avoid compensation, and this is particularly relevant in drug resistance cells, where redundant signalings are often present and amplified [11]. We initially found that different CK2 inhibitors sensitize MDR cells to vinblastin [58]. The administration of CK2 inhibitors in combination with conventional chemotherapeutics has been exploited in many other cases; in Table 2 we summarize the most relevant ones, where the efficacy has been assessed on resistant cells, or the synergistic effect has been confirmed in vivo.

**Table 2:** The more significant studies describing the effect of CK2 inhibitors in combination with drugs in resistant cells and/or in in vivo models

CK2 inhibitor	In combination with:	Tumor type	Resistance to:	In vivo study	REF
BMS-595 BMS-211	anti-CTLA-4 antibody	Lewis lung carcinoma, colon carcinoma, breast carcinoma		Yes	[120]
CGIB-300	Cisplatin	Cervical cancer		Yes	[75]
CX-4945	Cisplatin, carboplatin, gemcitabine	Ovarian cancer		Yes	[73]
CX-4945	Imatinib	Chronic myeloid leukemia	Imatinib		[17]
CX-4945	Vinblastin	T-lymphoblastic leukemia	MDR		[60]
CX-4945	Decitabine	Acute B-lymphoblastic leukemia		Yes	[121]
CX-4945	Gemcitabine/ cisplatin	Cholangiocarcinoma		Yes	[55]
CX-4945	Paclitaxel	Gastric cancer	Paclitaxel	Yes	[18]
CX-4945	Temozolomide	Glioblastoma		Yes	[122]
CX-4945	Fludarabine	Chronic lymphocytic leukemia	Fludarabine	Yes	[115]
CX-4945	Dabrafenib (BRAF inhibitor), erlotinib (EGFR) inhibitor)	Colon cancer	BRAF inhibitors		[123]
CX-4945	gefitinib/erlotinib	Lung cancer	gefitinib/erlotinib		[96]
CX-4945	MEK inhibitor AZD6244	Non–small cell lung cancer	EGFR inhibitor		[99]
CX-5011	Imatinib, MEK inhibitor U0126	Chronic myeloid leukemia	Imatinib		[16]
DRB	Doxorubicin	Cervical cancer		Yes	[19]
DRB, apigenin, emodin	TRAIL Anti-Fas	Endometrial carcinoma	TRAIL		[23]
Quinalizarin	Ionizing radiation	Lung cancer		Yes	[124]
Quinalizarin	Pim-1 inhibitor TCS	T-lymphoblastic leukemia	MDR		[119]
TBB	Imatinib	Chronic myeloid leukemia	Imatinib		[85]
TBB/IQA/2a	Vinblastin	T-lymphoblastic leukemia	MDR		[58]
tTBB (also known as TBBz)	HSP90 inhibitor 17-AAG	Multiple myeloma		Yes	[125]

In addition to kinase activity inhibition, the depletion of CK2 subunit(s) was found to enhance the sensitivity of human pancreatic cancer towards chemotherapeutic agents [126].

It is worth noting that resistance to CK2 inhibitors has been also found. Bian and colleagues observed modest antitumor efficacy of CX-4945 treatment in an in vivo model of head and neck cancer, with a concomitant compensatory increase of MEK/ERK/AP-1 pathway. The authors therefore suggest that combination with MEK inhibitors might overcomes CX-4945 resistance [127]. The same synergism (CX-4945 plus MEK inhibitor) was found effective in non–small cell lung cancer cells [99].

Interestingly, a study tested the potential for rapid emergence of resistance to CK2 inhibitors, and selected a TBB-resistant cell line that expressed a CK2 mutant; however, this line was sensitive to CX-4945 [24]. These results, if on one hand demonstrate that mutations within CK2 itself can emerge, causing resistance, on the other hand suggest that the problem can be tackled by combinations of CK2 inhibitors.

In the context of this paragraph on CK2 inhibitors, it might be interesting reminding that some of them have been derivatized to simultaneously target different molecules, as in the case of Cx-platin, a CK2 targeting Pt-based drug, able to reverse cisplatin resistance by causing DNA damage and inhibiting CK2-mediated DNA repair activity [31]. Moreover, the possibility exists for the development of molecules targeting CK2 and extrusion pump of the ABC protein family [61, 62]. Finally, although not pertinent to cancer, we would like to mention that, to counteract bacteria resistance to aminoglycoside antibiotics, CK2 inhibitors have been proposed as a structural base to design nucleotide-competitive inhibitors against aminoglycoside O-transferases [128].

## Conclusions

**Fig. 4:**
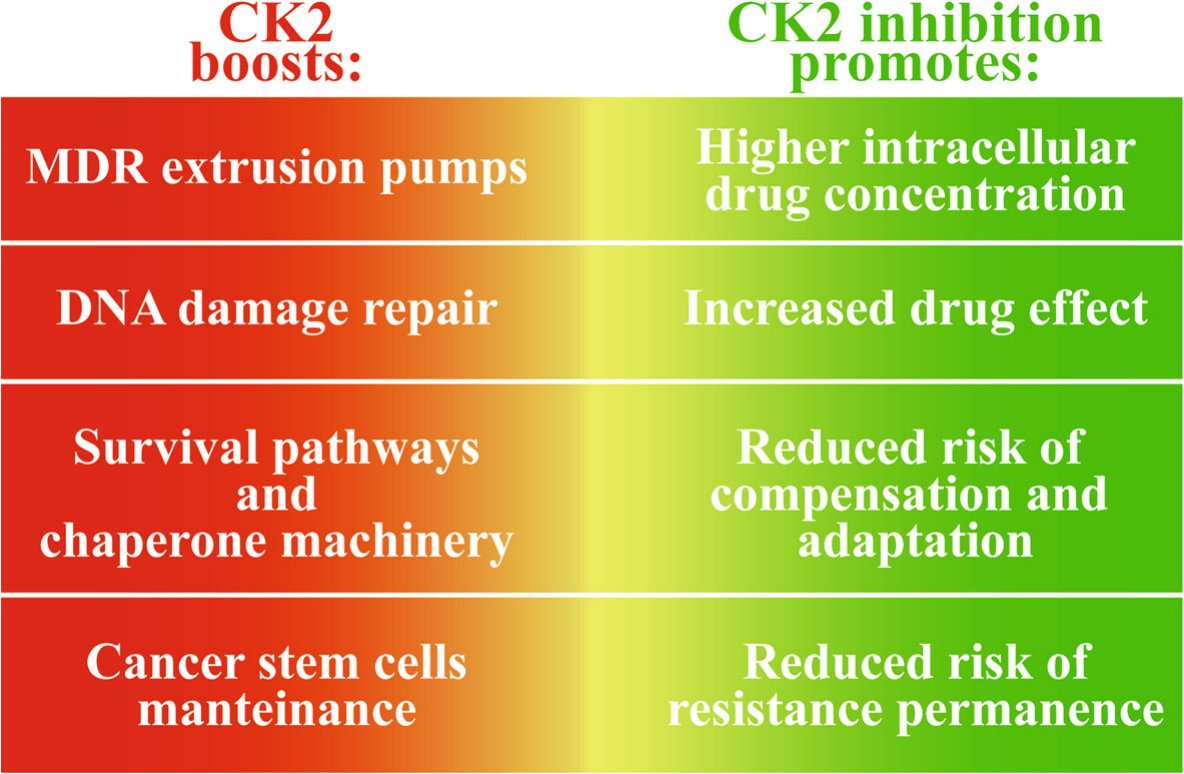
Summary of mechanisms of drug resistance potentiated by CK2 (left side, red), and corresponding effect of CK2 inhibition (right side, green)

This review underscores CK2 as an attractive target to counteract drug resistance in cancer. It plays roles at several crucial levels in chemo-resistance, ranging from the control of activity and/or expression of the major extruding pumps mediating MDR, to the DNA damage repair, the potentiation of survival signaling and cell protecting chaperone machinery, and the maintenance of cancer stem cells. Therefore, CK2 blockade might be advantageous for increasing intracellular drug concentrations, for allowing drug effects, and for preventing compensatory events (Fig. 4). A number of CK2 inhibitors have already been developed [111–113]. They have been proven effective in different types of cancer and drug resistant cells, and a study reports their action also in the presence of stromal cell [115]. Importantly, CK2 inhibitors are not recognized by the MDR extrusion pumps, and, alone or in combination with conventional anticancer drugs, they have produced encouraging results in several in vivo studies, supporting their future application in therapy.

As it could have been predicted, resistance against CK2 inhibitors has also emerged; however, due to the availability of structurally unrelated compounds, it appears that the problem can be easily overcome, as demonstrated by the sensitivity to CX-4945 of a TBB-resistant CK2 mutant developed during cell treatment [24].

In this promising scenery, we cannot omit to warn that, in some cases, caution should be taken while planning CK2 targeting. First, the combination of CK2 inhibitors with other drugs might not be convenient: in melanoma and thyroid carcinoma with wt BRAF, the effect of CK2 inhibition in combination with vemurafenib or selumetinib (BRAF/MEK inhibitors) was poor or even antagonistic [89]. Secondly, several evidences support a positive role of CK2 in allowing the cellular response to topoisomerase I-targeting drugs, suggesting that, in this case, combination therapy with CK2 inhibitors would be contraindicated [46–48]. Thirdly, it is debated whether responsiveness to CK2 inhibition requires p53 functions, which would imply the inadequacy of CK2 targeting in case of *TP53* mutation/deletion [38, 39, 83, 91]. Finally, the employment of chemical inhibitors might not always be the right strategy to target CK2, since also CK2 functions which are not dependent on its catalytic activity have been reported [18, 90]. All these observations suggest that the employment of CK2 inhibitors should be carefully planned for each specific circumstance, as indeed always occurs for the rational therapeutic drug combination in modern oncology.

In the framework of this review, it could be worth to mention that CK2 is also implicated in key processes which lead to radio-resistance: inhibition of CK2 has been found to reduce the secretion of IL-8 and IL-6 by endothelial cells after ionizing radiation (IR), and proposed as a strategy to improve the IR outcomes in non-small cell lung cancer cells [124, 129].

A final issue that might deserve few words is the CK2 implication in other types of resistance, as to insulin [130], antifungal drugs [131], and metal ions [132, 133], which are beyond the purpose of this review.

## Data Availability

Not applicable.

## Abbreviations

ARC: Apoptosis repressor with caspase recruitment domain
BCRP: Breast cancer resistance protein
BRAF: Serine/threonine-protein kinase B-raf
BRD4: Bromodomain-containing protein 4
BTIC: Brain tumor initiating cells
CK2: Protein kinase CK2, casein kinase 2, CK-II
CLL: Chronic lymphocytic leukemia
CML: Chronic myeloid leukemia
CSC: Cancer stem cell
CX-4945: 5-[(3-chlorophenyl)amino]-benzo[c]-2,6-naphthyridine-8-carboxylic acid
CX-5011: 5-[(3-ethynylphenyl)amino]-pyrimido[4,5-c]quinoline-8-carboxylic acid
D11: 1,3-dichloro-6-[(E)-((4-methoxyphenyl)imino)methyl] diben-zo(b,d) furan-2,7-diol
DMAT: 2-dimethylamino-4,5,6,7-tetrabromobenzimidazole
DSB: DNA double-strand break
EGFR: Epidermal growth factor receptor
EMT: Epithelial-mesenchymal transition
FHA: Forkhead-associated domain
FLIP: FLICE-inhibitory protein
GLI: Glioma-associated oncogene
GSI: γ-secretase inhibitors
HER2: Receptor tyrosine-protein kinase erbB-2
HMGA: High mobility group AT-hook protein
HSP: Heat shock protein
IKB: Nuclear factor kappa-B inhibitor
IKK: Inhibitor of nuclear factor kappa-B kinase
IR: Ionizing radiation
JWA: ADP-ribosylation factor-like protein 6-interacting protein 5
KRAS: GTPase KRas
MDR: Multidrug resistance
MRE11: Meiotic recombination 11 homolog 1
MRN: MRE11-RAD50-NBS1 complex
MRP1: Multidrug resistance-associated protein 1
MTT: 3-(4,5-dimethylthiazol-2-yl)-2,5-diphenyltetrazolium bromide
NBS1: Cell cycle regulatory protein p95
NF-κB: Nuclear factor kappa-B
NHEJ: Nonhomologous end-joining
p53: Tumor suppressor p53
P-gp: ATP-binding cassette sub-family B member 5
PI3K: Phosphoinositide 3-kinase
PML: Promyelocytic leukemia protein
PTEN: Phosphatidylinositol 3,4,5-trisphosphate 3-phosphatase and dual-specificity protein phosphatase
PXR: Pregnane X receptor
RAD50: DNA repair protein RAD50
RXR: Retinoid X receptor
SHH: Sonic hedgehog
T-ALL: Acute T lymphoblastic leukemia
TBB: 4,5,6,7-tetrabromobenzotriazole
TBBz or tTBB: 4,5,6,7-tetrabromo-1H-benzimidazole (also known as TBI)
TCF/LEF: T-cell factor/lymphoid enhancer-binding factor
TKI: Tyrosine kinase inhibitors; Topo: topoisomerase
TRAIL: TNF-related apoptosis-inducing ligand
XRCC: X-ray repair cross-complementing protein
